# The role of ambidextrous leadership and employee ambidexterity in enhancing service quality of care and creativity – a study of health professionals

**DOI:** 10.1186/s12913-023-10275-3

**Published:** 2023-11-14

**Authors:** Terje Slåtten, Barbara Rebecca Mutonyi, Anne Jørgensen Nordli, Gudbrand Lien

**Affiliations:** 1 Inland School of Business and Social Science, Inland Norway University of Applied Sciences, Campus Lillehammer, Lillehammer, 2604 Norway; 2 Kristiania University College, Oslo, Norway

**Keywords:** Ambidextrous leadership, Employee ambidexterity, Exploration, Exploitation, Service quality of care, Creativity, Health professionals, Home-care services

## Abstract

**Background:**

This study aims to empirically examine the role of ambidextrous leadership on employees’ ambidexterity and job-directed performance. Ambidextrous leadership encompasses a leader’s capability to stimulate exploitative and explorative activities in employees. Specifically, the study explores in detail how ambidextrous leadership is linked to two types of job-directed performance in health professionals, namely service quality of care and creativity, in addition to what role employee ambidexterity has in this relationship.

**Methods:**

A cross-sectional survey was developed, and data were gathered through convenience sampling of *N* = 258 health professionals of in-home care services across municipalities based in Norway. The study’s conceptual model was analyzed through structural equation modeling partial least squares with SmartPLS 3 software. Mediation by Bootstrap was used to analyze the indirect relationships.

**Results:**

Ambidextrous leadership was found to have a direct impact on both employee service and quality of care ($\beta$ = 0.236) and employee ambidexterity ($\beta$ = 0.395). The direct relationship between ambidextrous leadership and employee creativity was nonsignificant. However, the relationships between ambidextrous leadership and service quality of care and creativity were both mediated by employee ambidexterity. Finally, the results reveal that employee creativity mediated the relationship between employee ambidexterity and service quality of care.

**Conclusions:**

The results show that ambidextrous leadership and employee ambidexterity promote the job-directed performance of health professionals. Thus, a practical implication is that health-care organizations should recruit, train, and develop their leaders to become ambidextrous leaders, in addition to being aware of the multiple direct and indirect effects of practicing ambidextrous leadership. Doing so will have a direct positive impact on the level of service quality and employee ambidexterity.

## Background

Leadership matters in all types of organizations; specifically, “leadership is important to quality …in healthcare organizations” [1, p. 1]. As such, it is crucial to further our current understanding of the various impacts of leadership practice in health-care organizations. First, this study explores the role of ambidextrous leadership practice and how it is linked with health professionals’ service quality of care (SQC). Ambidexterity is about managing and balancing explorative versus exploitative practices, either as a leader or an employee. Second, the study examines whether and how ambidextrous leadership is capable of having an impact on more indirect aspects related to health professionals’ SQC. This latter aspect is manifested in the ambidexterity and creativity (C) of the employee.

Undoubtedly, leadership has an impact on numerous aspects of an organization, including its individual members. Most of us probably have some experience with “good” or “bad” leadership practice and, respectively, how it positively or negatively influences and affects our thoughts, emotions, and activities, and how it can even have an impact on job performance (e.g., service quality, productivity, among other aspects). Clearly, organizations need to understand the role of leadership practice.

Specifically, this paper focuses on health professionals defined as health workers with a role at the “forefront” of in-home care services [2]. Health professionals’ from in-home care services, which are similar to most other services offered by health-care organizations, are characterized by a high level of human factors and the involvement of people-intensive organizations [2]. In 2020, in Norway (where this study is undertaken), 146,000 people were employed in in-home care services [3]. Because health professionals work “face to face” with the “customers” (most often termed “users”), they are, in many ways, considered to be the main ones responsible for the SQC provided by the health organization.

Two aspects make it potentially challenging to deliver an excellent level of service quality to users of in-home care services. First, the content of services offered to users varies greatly. It includes providing users with medical help, support in daily life activities, rehabilitation, and nursing care [2]. Second, all services from health professionals are given (only) at the users’ homes. Thus, the type of service is reflected in the name, i.e., “home care services.” In combination, considering the context of this study (Norway), these two aspects imply that health professionals have to be capable of mastering and executing various tasks and sometimes offering services at very distant places and districts.

Consequently, the work role of health professionals is relatively embedded with complexities and challenges. Because of this, there is a need for managers of health-care services to perform a leadership style and daily leadership practice that, as much as possible, help health professionals “to think and act” in such a way that they become excellent “frontline” providers of SQC to users of in-home care service. It is reasonable to assume that in such complex work contexts, leadership practices that are both explorative and exploitative may be more appropriate and thus contribute to increasing health professionals’ service quality. A recent review by Vaughn et al. indicates the importance of appropriate leadership practice for service quality. The authors found that organizations that struggled to improve quality were characterized by disconnected leadership [4]. Clearly, leadership practices are essential to enhance health professionals’ job performance (e.g., service quality).

Moreover, given the complex and challenging workdays that health professionals face, creativity and ambidexterity among the employees may also be important [4], which is why this study also investigates the role of these concepts, i.e., what they mean for service quality, and how employee ambidexterity may act as a mediator for leadership effects. C may enable health-care professionals to think outside the box and develop innovative solutions to address the unique needs of each user (similarly to the bricolage innovations introduced by Fuglsang [5]). Employee ambidexterity may, in addition, allow health professionals to balance the demand of providing efficient and reliable care while also adapting to dynamic and unforeseen situations [6]. For instance, it can manifest as switching between structured routines and flexibly responding to changing clients’ needs or emergencies. Thus, since the employees are key players in delivering high-quality services [4], it is also reasonable to assume that employee ambidexterity may act as a mediator for how ambidextrous leadership affects service quality.

Based on the above considerations, two important aspects shaped the aim and focus of this study. First, according to Ree and Wiig, there is a knowledge gap regarding quality issues when one considers studies undertaken within a home care context [6], especially in Norway. Second, there seems to be scarce research that examines the role of leadership practice and SQC within an in-home care service in a Norwegian context. One exception is the study of Ree [1], in which the author, among other factors, examined how transformational leadership was linked to health-care personnel’s perception of quality regarding providing person-centered care to the user of in-home care services. Except for the study of Ree [1], it seems that very few studies on in-home care service in a Norwegian context have focused on quality improvement with a focus on factors associated with leadership intervention [e.g., 7, 8]. Although these studies are undoubtedly interesting, there is a need for more substantial research that examines the role of leadership style for SQC from the perspective of health professionals at the “front” of offering in-home care services to users.

Given the knowledge gap in previous research, this study has two overall aims. First, we explore the role of ambidextrous leadership practice and how it is linked with the SQC of health professionals. Second, we examine whether and how ambidextrous leadership is capable of having an impact on more indirect aspects related to health professionals’ SQC. This latter aim is manifested in employee ambidexterity and employee creativity. Rooted in a literature review of the concepts, a conceptual model is developed and further analyzed with Partial Least Square – Structural Equation Modeling (PLS-SEM).

The study responds to a recent call to further research on ambidextrous leadership within a health-care context [9]. To the best of our knowledge, this is a pioneering study that conceptualizes and empirically examines factors associated with ambidextrous leadership as well as employee ambidexterity within health services research.

The paper is arranged as follows. First, the concepts of interest are defined, and linkages between them are discussed. Second, hypotheses are presented, and a conceptual model is depicted. Third, the methodology, description of the chosen context, and results from the empirical study are reported. Fourth, this section discusses the findings and proposes suggestions for further research based on this study. The paper ends with a conclusion.

## Review of the literature

**Fig. 1:**
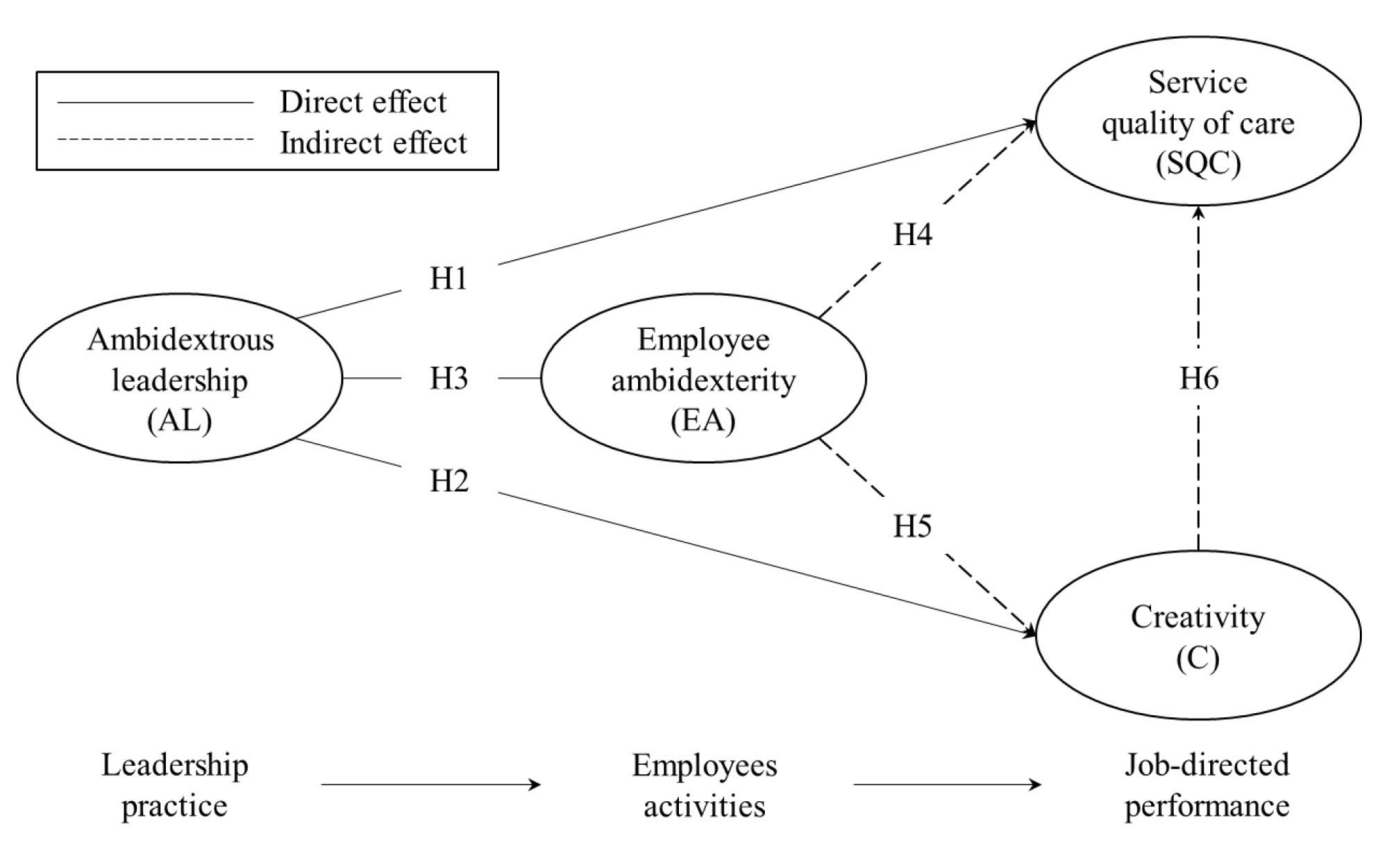
Conceptual model

The first part of this section describes the concept from Fig. 1, the conceptual model, which is depicted in the next section. The presentation of individual concepts follows a logic of relationships, starting from the left side of Fig. 1 (with “leadership practice”) to the right side. The second part of the section considers the relationships between the different concepts.

### Ambidextrous leadership (AL)

Leadership style is suggested to be a key factor in influencing employees’ attitudes and behaviors [10]. Consequently, good reasons exist to focus on how leadership practice affects health professionals. In this study, we limit our focus to a leadership practice termed AL. The word “ambidexterity” “literally means the ability to use both hands with equal ease” [11 p. 957]. Consequently, ambidexterity is about the capacity of someone (e.g., a leader) to combine a duality of features very distinct from each other.

In this study, AL embraces health professionals’ perception of whether the practice of their leader was demonstrated through a combination of the duality between two dimensions, namely (i) “*opening*” and (ii) “*closing*” leadership behaviors [10]. Consequently, AL consists of two dimensions.

The first dimension that constitutes AL, opening behavior, is a leadership practice directed toward actively stimulating and encouraging explorative activities among employees (e.g., health professionals). Rosing et al. list several examples of opening leadership behavior, such as a leadership practice of “allowing different ways of accomplishing a task, encouraging experimentation with different ideas, motivating to task risks, giving possibilities for independent thinking and acting, giving room for own ideas, allowing errors, encouraging error learning” [11, p. 967]. The fundamental idea of opening leadership behavior is to trigger employees to break out of existing and sometimes potentially ingrained routines, and stimulate them to think in novel ways.

The second dimension that constitutes AL, closing behavior, is diametrical to opening behavior. Closing leadership practice is directed toward actively stimulating and encouraging exploitative activities among employees (e.g., health professionals). Examples of closing leadership behavior include “monitoring and controlling goal attainment, establishing routines, taking corrective action, controlling adherence to rules, paying attention to uniform task accomplishment, sanctioning errors, sticking to plans” [11, p. 967]. The focus in closing leadership behavior is in contrast to opening leadership, to decrease the disparity in employee behavior, and to standardize to promote consistency and make behavior more or less uniform.

In line with the meaning and content of the word “ambidexterity,” it is important to note that although “opening” and “closing” leadership behavior are clearly two separate dimensions of AL, it is the combination that constitutes AL. Thus, in line with previous research, AL promotes “both explorative and exploitative behaviors in followers by increasing or reducing variance in their behavior and flexibly switching between those behaviors” [11, p. 957].

How much a leader should either increase or reduce their opening and closing behavior as a part of their AL is dependent on the specific situation, the task to be solved, and potentially other conditions that should or could be relevant to consider. In line with this, Usman et al. noted that “the multifaceted work settings necessitate the display of both opening and closing behaviors at a time or switching between these two behaviors” [12, p. 2]. Consequently, the adjustment of and what is the right combination of AL (referring to opening and closing behavior) will vary. Therefore, Rosing et al. suggest that “these behaviors [referring to opening and closing behavior] need to be shown ad hoc” [11, p. 967].

It is worth noting that this study has no specific attention and focus directed toward assessing the exact balance or uncovering individual differences in the composition of the two dimensions of AL. This study limits its focus to only emphasizing the total “sum of the two dimensions” of AL.

As visualized in Fig. 1, the focus is on the role AL seems to have in enhancing health professionals’ SQC and C, and what role employee ambidexterity seems to have in this relationship is also examined. This is discussed in the following sections.

### Service quality of care (SQC)

SQC is categorized as one of two types of job-directed performance of health professionals (Fig. 1). It seems that the content of the concept of (service) quality within the domain of health service research varies greatly depending on what type of role one has in a health-care organization [13], such as whether it is considered from, for example, the perspective of a manager or health-care professional. However, a potential “solution” to this “problem” is to adopt a definition and perspective of (service) quality that can be judged to be the most appropriate and considered as best to match the organizations and type of role one possesses in the specific health-care organization studied.

In this study, the focus is on health professionals possessing a role at the “front” and thus working closely “face to face” with users, offering them various home care services. In a recent qualitative study by Aase et al. [14], the authors explored, among several other aspects, how the meaning and content of the word “quality” was understood among health-care professionals in in-home care (and thus comparable with the focus in our study). One of the conclusions based on their study was that quality among health-care professionals should encompass a patient-centered approach to care. In line with this, the authors state the importance of understanding “people’s experience of quality to succeed in quality improvement interventions” [14, p. 9].

Based on these ideas of how to best capture what “quality” means, in this study “quality” is approached from the perspective of health professionals at the “front” in in-home care. Specifically, “quality” refers to health professionals’ experience and judgment of their overall service provided to in-home care users and is termed SQC. Consequently, SQC embraces health professionals’ holistic perception of the service offered to in-home care users. This approach to study and define service quality from a frontline employee perspective has been widely used in previous studies both within health-care [e.g., 15, 16] as well as within service quality literature in general [17]. Previous studies have suggested a relatively “psychological closeness” between providers’ assessment and receivers’ assessment of service quality [18]. Consequently, it is reasonable to assume that studying SQC as done in this study is appropriate.

In this study, it is assumed that AL can have an impact on SQC. Because a leader possesses a formal authority to lead others, they have the necessary means, through their role position, to have power and an impact on members of an organization [19, 20]. Specifically, AL may give health professionals the necessary autonomy and motivation to develop further and improve their SQC offered (referring to the “opening” dimension of AL) simultaneously as they focus on making consistency and refining their SQC (referring to the “closing” dimension of AL). Thus, those leaders that involve and engage themselves in AL by combining “opening” and “closing” leadership behaviors will have a positive impact on health professionals’ SQC.

Previous research has suggested that leadership practice is linked to the provision of quality care in health-care organizations [1] and is essentially a factor in fostering both attitude and behavior of employees [10]. The assumption about a link between AL and SQC also finds support within the job demands-resources (JD-R) framework [21]. Based on this JD-R framework, when considered as a form of positive form of (leadership) support, AL will have a positive impact on employee outcomes, such as SQC health professionals. Considering the abovementioned rationale, several good reasons exist to expect AL to be positively associated with health professionals’ SQC. However, no previous research has empirically examined what role AL has for SQC in the health-care context. Thus, this study contributes to closing a relevant and important knowledge gap. This relationship can be formally expressed in the following first hypothesis:

#### Hypothesis 1

*There is a positive relationship between health professionals’ AL and their SQC.*

### Creativity (C)

Creativity (C) is categorized as the second type of job-directed performance of a health professional (Fig. 1). In this study, C refers to health professionals’ cognitive thinking skills. It focuses specifically on a person’s mental capability to develop and generate novel and useful ideas to solve problems and/or improve how things are done [22]. Although the two are closely related, a person’s C differs from its innovative performance. While creativity is a cognitive concept (thinking capability), innovative performance is a behavioral concept (behavioral capability). It can be described as the visible manifestation of creativity into practice and action (e.g., a behavioral change in how services are provided to patients). Thus, innovation is a function of C, where the latter acts as fuel and a necessary input to innovation [9, 23]. For example, Fuglsang [5], who investigates bricolage innovations in the home-care context, refers to the process of creating new solutions or approaches by utilizing available resources flexibly and adaptively, and by creatively recombining existing resources, tools, and knowledge to address emerging challenges and generate innovative solutions.

C is a relevant capability, especially for health professionals who work on the “front” with users and patients. According to Hewko, “Without engaging in …creative thinking, professionals may find it difficult to identify what, where, and how new ways of working (i.e., innovations) can be introduced” [24, p. 1]. Consequently, because of their close contact with and relationship with users, the C of health professionals is a desirable “mental tool” that helps them to suggest necessary changes and reveal areas of improvement in the patient care and services offered.

The literature suggests that leaders of health-care organizations should be interested in stimulating and promoting health professionals’ C. Patterson and Zibarras [25] stress the significance of C because of the instant shifts in health care. The authors suggest that more focus on employee’s C is needed. In line with this, they state: “There is an increasing need for individuals to generate new ideas [and aspect of creativity] and implement these to improve working practices” [25, p. 418].

Although the literature within health services research suggests that it is important to focus on the C of a health professional, surprisingly, little research has empirically examined how and what types of leadership styles can positively enhance creativity. There is an example of how leadership styles, such as leadership autonomy support [9], are linked to health professionals’ C, but no previous study has examined whether and how AL is capable of promoting the C of health professionals. However, there are good reasons to expect to find an association between AL and C. First, as also noted previously in this paper, leadership is one of the most critical factors to influence and impact employees in organizations [10, 19, 20]. Second, the nature of what is embraced in AL provides reasons to assume that AL can develop the C of health professionals. For example, the AL labeled “open” allows employees in the organizations to experiment and develop their ideas and independent thinking [11], which are all associated with leadership behaviors that potentially foster and expand the creative thinking skills of employees. In line with this assumption, Mohiya and Sulphey noted: “Ambidextrous leaders…encourage their subordinates to proactively identify innovative ideas [which is equivalent to being creative] and solutions in the workplace” [26, p. 3]. Third, although studies have been undertaken outside the health service context, previous research supports a positive association between AL and C, including student’s C, as in the study by Antonio et al. [27]. Consequently, based on the three arguments mentioned above, iAL is expected to be positively related to health professionals’ C. This reasoning leads to the following second hypothesis:

#### Hypothesis 2

*There is a positive relationship between health professionals’ AL and their C.*

### Employee ambidexterity (EA)

EA is categorized as “employees-activities.” Placing EA in the middle of Fig. 1 indicates that EA is supposed to act as a mediator between AL (“leadership practice”) and the SQC and C (“job-directed performance”).

The concept of EA is relatively comparable to the concept of AL in two ways. First, both concepts focus on ambidexterity reflected in combining a duality that is very distinct from each other [11]. Second, EA and AL are similar, as both concepts focus on a person’s behavioral orientation toward the (two) contradictory activities embraced in ambidexterity. On the other hand, there is one clear and major difference between the two (EA and AL) concepts. In other words, instead of studying ambidexterity as a leadership practice (referring to AL), the focus in EA is shifted to studying how ambidexterity is reflected in employee practices. It is important to note that this implies that the scope of what is labeled “employee’s activities” in Fig. 1 in this study is limited and narrowed to only focus on the combination of those (two) activities embraced by EA.

Taking into account the scope and focus mentioned above, EA in this study refers to “behavioral orientation of employees to combine exploitation and exploration associated activities” [28, p. 3]. Based on ideas from the original work of March [29], the conceptualization of both exploration and exploitation in EA is adapted to the frontline health professionals in this study. Exploration activities are “things captured by terms such as search, variation, risk taking, experimentation, play, flexibility, discovery” [29, p. 71]. On the other hand, exploitation activities are described as “refinement, choice, production, efficiency, selection, implementation, execution” [29, p. 71].

Based on this idea, exploration activities in this study are understood as “behaviors related to experimentation, searching for alternative ways to accomplish a task, and learning from errors” [30, p. 697]. Furthermore, exploitation activities are understood as “relying on previous experience, putting things into action, and incrementally improving well-learned actions” [30, p. 697]. When comparing and contrasting the duality of the two types of behavioral activities of EA, it becomes clear that they are divergent, contrary, and paradoxical. While exploitation activities are about trusting well-established rules and routines, improving the exploration activities is all about breaking out well-established rules and routines, actively seeking to create variation and intentions to do something new.

Although the two behavioral activities of EA are different, they should not be studied separately. In line with the meaning of the word “ambidexterity,” it is (only) the combination and sum of the two behavioral activities (referring to exploration and exploitation activities) that represents EA.

It is realistic to assume that “how much” EA an individual possesses will vary among members of an organization. Thus, EA is heterogeneous and distributed across employees of an organization. Furthermore, in specific situations or periods, an individual can be mostly oriented toward one activity of EA, such as, for example, exploration activities, and less oriented toward exploitation activities. This implies that in some cases, one behavioral activity of EA will dominate over the other. Consequently, the “sum of” EA of individuals is not static or fixed but will most likely change across time, situations, contexts, and work tasks. This assumption of EA is in line with how ambidexterity is described in the literature. Specifically, EA is not described as a psychological trait of a person that is relatively fixed, static, and unchangeable. As Alghamdi remarked: “Instead of being a psychological trait, ambidexterity of an individual is an individual behavioral capability” [28, p. 4]. Thus, EA as a “behavioral capability” suggests that the EA of an individual is dynamic, manageable, “controllable,” and potentially changeable in a specific direction if necessary or desirable.

It is important to “create a context that allows individual employees to act ambidextrously” [30, p. 696]. In this study, it is assumed that an organizational context where leaders are practicing AL can encourage EA positively. There are several good arguments about why one should expect a positive association between AL and EA. First, as stated in the above, leadership practices are the key factor in motivating and influencing employees’ attitudes and behaviors in organizations [10]. Second, AL and EA embrace a similar focus on the behavioral orientation manifested in either leaders’ or employees’ exploration and exploitation activities. Based on their similar focus, and since leaders and employees share the same context, it is reasonable that leaders practicing AL will engage employees to adopt a similar pattern of behavioral orientation. Consequently, there will be a transmission effect from leadership (AL) behavioral orientation to employee behavioral orientation regarding exploration and exploitation activities (referring to EA). Third, according to Alghamdi, EA “can be boosted via any factor that increases exploration and exploitation” [28, p. 4]. Because leaders possess the power and formal authority to lead and simultaneously act as role model organizational members, there are good reasons to expect AL to positively “boost” EA. The idea of transmission from AL to EA is also supported by social learning theory [31]. This theory posits that others (e.g., employees) learn what is “right” and “wrong” behavior by observing others (e.g., leaders). Fourth, to the authors’ knowledge, no studies have been done in a health-care context. However, a few previous studies support a positive association between AL and EA. For example, in their study of public employees in public legal service agencies, Luu et al. [32] found empirical support for a positive relationship between AL and frontline public EA. In a similar vein, Alghamdi [28], studying university faculty members, found support for a positive relationship between the “ingredients” of AL (referring to opening and closing leadership behaviors) and the “ingredients” of EA (referring to exploration and exploitation employee behaviors). The lack of such studies in the health-care context underlines the knowledge gap that this study contributes to.

Based on the arguments mentioned above, there are good reasons to expect to find the same relationship pattern between AL and EA in this study of health professionals. Thus, the following hypothesis is proposed:

#### Hypothesis 3

*There is a positive relationship between the AL of health professionals and their EA.*

In the first two hypotheses, it was suggested that AL directly impacts SQC and C. There are good reasons for thinking that AL has such a direct role. However, in addition, as also visualized in Fig. 1, it is also reasonable to postulate that EA plays a mediating role between these suggested variables. This presumes EA to function as an alternative and complementary “route” of how AL works to influence the SQC and C of employees compared to what was discussed in hypotheses 1 and 2.

The main argument for suggesting EA as a mediator is because both employees’ SQC and C (“job-directed performance”) is a consequence or a “product” of those intertwined cognitive (thinking) processes and behavioral activities stemming from employees themselves. This suggests that although it is important for health professionals to deliver high SQC, it is equally important to act creatively, implying the importance of health professionals’ EA [9, 14, 15, 24]. In this study, these intertwined thinking and behavioral activities are embraced in the context of EA. Specifically, a leader practicing AL is limited to only facilitating and motivating employees to improve their SQC and enhance their C. At the same time, the employees themselves must do the SQC and C “work.” Moreover, it is only when employees are motivated to engage in thinking and behavioral activities (referring to EA) that positive changes in SQC and C can be manifested in reality and action. This implies that EA mediates AL, SQC, and C. This expectation about EA as a mediator leads to the following two hypotheses:

#### Hypothesis 4

*The relationship between health professionals’ AL and SQC is mediated by EA.*

#### Hypothesis 5

*The relationship between health professionals’ AL and C is mediated by EA.*

In the previous discussion, C was discussed as input or fuel necessary for innovation [9, 23]. However, in this study, we expand the usefulness and value of developing employees’ C to include its impact on SQC. Specifically, it is assumed that the C of employees working in the “front” (referring to health professionals) could also function as a necessary input or fuel to undertake new and positive changes in their SQC offerings to “customers” (referring in this study to users of in-home care). These changes in the SQC of employees (e.g., new changes in procedures, processes, and types of services offered) can be compared with what is embraced in the meaning of innovation. The argument for this assertion is simple. In its pure nature, innovation can be described as doing something new to undertake and implement new changes compared to what has been done in the past.

New changes can practically take place in every organization, including the work of employees at the “front.” The “newness” of changes can vary from minor or incremental to major or radical. Because employees in the “front” have direct contact, and sometimes close contact with “customers” over a relatively long period, these employees are uniquely positioned to use their C to identify new changes that will enhance the customers’ experience of their SQC. Consequently, employee engagement in C is a prerequisite to positive changes or improvements in SQC. This idea is supported by Hewko, who states, “Without engaging in… creative thinking, professionals… find it difficult to identify… new ways of working” [24, p. 1].

However, it is not reasonable to assume that employee engagement in C exists independently and is under a perpetuum mobile. On the contrary, and most likely, employees’ C must be stimulated, triggered, and actively driven by someone. As proposed above, EA, stemming from AL, is suggested as one such source that is capable of stimulating or triggering and engaging employees’ C. This reasoning implies, as visualized and suggested in Fig. 1, a linkage or “domino effect” between EA and SQC via employees’ C. Specifically, it assumes that when employees’ C increases, because of a positive shift in their EA, this will lead to an improvement in the experience of SQC. Consequently, employees’ C is assumed to mediate the association between EA and SQC. The following hypothesis summarizes the final assumption about the relationship proposed in this study:

#### Hypothesis 6

*The relationship between health professionals’ EA and SQC is mediated by C.*

To the authors’ knowledge, no studies in the health-care context have until today addressed the roles of EA and C that were argued in hypotheses 4, 5, and 6, which were further investigated in this study. It is thus assumed that the study contributes important knowledge to the field.

## Conceptual model

Figure 1 depicts the conceptual model. All concepts in Fig. 1 are categorized into one of three groups, labeled (i) “Leadership practice,” (ii) “Employee activities,” and (iii) “Job-directed performance.” The arrow signals the presence of a relationship between each of the three groups. Specifically, starting from the left in Fig. 1, we assume a logic where leadership practice is expected to function as a triggering factor that has an impact on the other two (“Employees’ activities” and “Job-directed performance”).

In line with the aim and focus of this study, AL represents the category “Leadership practice.” EA represents the category “Employee’s activities.” SQC and C represent the category “Job-directed performance.” The solid line in Fig. 1 visualizes the assumption about direct relationships, while broken lines visualize the assumption about indirect relationships.

## Methods

### Sample and procedure

Previous research has found that promoting health professionals’ job performance strengthens the overall efficiency and the quality of patient care in health-care organizations [13, 14]. This entails that leadership, specifically the type of leadership, plays a crucial role in fostering health professionals’ job performance [1]. In detail, this study has empirically focused on two types of job performance, namely SQC and C. As various types of leadership have been found to influence health professionals’ job performance [e.g. 1], the call for further knowledge in this area remains valid [6–8]. Therefore, the study has specifically focused on empirically exploring the impact of AL on health professionals’ job performance and, as such, sampled health professionals in home-care institutions. Approximately 17 health-care service institutions in Inland Norway were invited to participate in the project. Out of the 17, nine willingly agreed to participate in the project, an acceptance rate of approximately 53% from home-care institutions, reflecting geographical differences to obtain generalizable results that potentially represent the targeted population. Inland Norway was chosen because the project leader was affiliated with the county’s home-care office. For selecting respondents, the initial contact and all contacts with the in-home care service institutions were sought by and through the health organizations’ Director of Knowledge and Development (DKD). The DKD would forward information to department heads, who then would forward it to their employees. The health professionals were invited to the study and informed of its purpose, the procedures for anonymity, and that the participation was voluntary. Before gathering data, several pre-tests were performed, both by a selected group of health professionals and three experts who reviewed the claims and performed back-to-back translations of the claims. Thus, claims were translated from English to Norwegian and back to English. It is important to note that the Norwegian health-care service employees adopted the claims. After revisions, the link to the final survey was forwarded to the DKD, which sent the information to the department heads, who in turn informed and invited their employees to participate. Note that the same procedure was followed in sending a reminder for participation. The survey was distributed online through a platform called Nettskjema (www.nettskjema.no). The choice of Nettskjema relied heavily upon the unique service that enables automatic deletions of IP addresses once a participant completes the survey. Thus, total anonymity could be ensured. Before the commencement of the survey, all participants were asked to consent by ticking off a box and “next.” This ensured that all were informed and consented to participate in the study voluntarily. The survey was distributed to about 500 employees, yielding 258 completed questionnaires, a response rate of 51.6%. The personal characteristics of the participants of this study are presented in Table 1.

**Table 1:** Personal characteristics of the study sample (*N* = 258)

		%
Staff role	Nurse	34.5
	Health professional	49.2
	Other (health professionals (bachelor), unskilled)	16.3
Employed	< 5 years	35.7
	6–15 years	26.7
	16–25 years	23.3
	> 25 years	14.3
Work hours	Part time	66.7
	Full time	33.3
Age	< 35 years	27.5
	35–50 years	38.4
	> 50 years	34.1

### Instruments

The conceptual model of this study employed well-established scales in examining the direct and indirect effects of AL on the health professionals’ job performance. As mentioned, the claims used in this study went through a back-to-back translation process. There are two reasons for this: first, to accommodate the local language needs of the participants, who mostly spoke Norwegian as their first language; second, to ensure that the claims in Norwegian are fit to the purpose of this study, as well as the context of this study. Claims in each main measure (AL, EA, SQC, and C) were adopted. Each measure included claims with the seven-point Likert scale, ranging from “strongly disagree” (1) to “strongly agree” (7). In addition, the survey included a section on demographic characteristics, as summarized in Table 1.

AL was adapted and measured through an 11-item scale from Roosing et al. [11] and Zacher and Rosing [33]. The original scale utilized a five-point Likert scale. This study employed a seven-point Likert scale based on the feedback from the pre-test of the survey. EA was adapted and measured through a six-item scale from Mom et al. [34]. SQC was adapted and measured through a three-item scale from Slåtten and Lien [20]. C was adapted and measured through a two-item scale from Zhou and George [35]. Table 2 summarizes all claims and their respective construct used in this study. It is important to note that the claims used in this study are part of a bigger research project.

**Table 2:** Constructs and claims used in the study

Construct	Claims label	Claims
	In general, my leader:	
AL	AL1	Allows different ways of accomplishing tasks.
	AL2	Encourages experimentation with different ideas.
	AL3	Gives possibilities for independent thinking and acting.
	AL4	Gives room for other colleagues’ own ideas.
	AL5	Allows errors.
	AL6	Encourages error learning.
	AL7	Monitors and controls goal attainment.
	AL8	Establishes routines.
	AL9	Takes corrective action, if necessary.
	AL10	Controls adherence to rules.
	AL11	Sticks to plans.
	In general, I engage in:	
EA	EA1	Searching for new possibilities with respect to my work.
	EA2	Focusing on strong renewal of services or working processes.
	EA3	Activities requiring me to learn new skills or knowledge.
	EA4	Activities in which I have accumulated a lot of experience.
	EA5	Activities that I clearly know how to conduct.
	EA6	Activities I can properly conduct using my existing knowledge.
	To which extent do you agree or disagree with these claims?	
SQC	SQC1	The assistance I provide is of high quality.
	SQC2	I provide assistance with a service minded manner.
	SQC3	My opinion is that I give my very best in all aspects of my work.
	To which extent do you agree or disagree with these claims?	
C	C1	In my job, I come up with creative solutions to problems.
	C2	In my job, I suggest new ways to increase quality.

### Data analysis

In this study, we employed partial least squares structural equation modeling (PLS-SEM) [36] as the data analytical procedure to test the hypotheses in our conceptual model using the SmartPLS 3 software [37]. In line with Hair et al. [36], PLS-SEM was chosen to analyze the conceptual model because this study focuses on explaining the variance in the conceptual model’s dependent variables (i.e. EA, SQC, and C). SmartPLS, as a software, is currently the primary software for variance-based use in PLS-SEM [36, 37]. PLS-SEM is based on a two-stage approach. In the first stage, the focus is on the reliability and validity of the item/claim measures used (measurement model). By contrast, the emphasis is put on the results from estimations of the part coefficients (structural model) in the second stage. Based on the PLS-SEM results, mediator effects were estimated and analyzed using the bootstrapping test of Zhao et al. [38]. The results of this study are reported according to the recommendation of Hair et al. [39] on reporting PLS-SEM data.

## Results

### Measurement model

As the measurement models contained only reflective constructs, the assessment was based on (1) convergent validity (the extent to which a variable is positively correlated with alternative variables used to measure the same construct, i.e. loading and average variance extracted); (2) internal consistency reliability (the magnitudes of the intercorrelations of the observed variables, using the criterion’s composite reliability and Cronbach’s alpha); and (3) discriminant validity (the extent to which a construct is distinct from other constructs, using the heterotrait–monotrait ratio criterion). As a minimum measurement quality standard, we used the ‘rule of thumb’ criteria by Hair et al. [36]. The results in Table 3 indicate that we have reliable and valid measurement models.

**Table 3:** Results of the measurement model for the AL, EA, SQC and C constructs ^*^ AVE = Average variance extracted; HTMT = Heterotrait–monotrait ratio of correlations

		Convergent validity		Internal consistency reliability		Discriminant validity
Construct	Claims label	Indicator reliability	AVE^*^	Composite reliability	Cronbach’s alpha	HTMT criterion^*^
‘Rule of thumb’		Loading > 0.7	> 0.5	0.7–0.95	0.7–0.95	HTMT interval does not include 1
AL	AL1	0.80	0.60	0.94	0.93	Yes
	AL2	0.85				
	AL3	0.72				
	AL4	0.87				
	AL5	0.70				
	AL6	0.81				
	AL7	0.66				
	AL8	0.80				
	AL9	0.83				
	AL10	0.75				
	AL11	0.70				
EA	EA1	0.79	0.70	0.93	0.91	Yes
	EA2	0.82				
	EA3	0.83				
	EA4	0.90				
	EA5	0.85				
	EA6	0.82				
SQC	SQC1	0.83	0.72	0.88	0.80	Yes
	SQC2	0.91				
	SQC3	0.80				
C	C1	0.96	0.94	0.95	0.93	Yes
	C2	0.97				

In addition to evaluating the measurement model, and in line with Podsakoff et al. [40], we also used the Harman single-factor test to evaluate common method bias. Harman’s single-factor test is one of the most widely used statistical remedies to minimize, if not potentially eliminate, issues of common method bias [40]. The results^1^ show that the eigenvalue is 7.81. With 22 items (see Table 2), we get 7.81/22 = 0.35, which means that 35% of the total variance is explained by a factor that is far below the 50% “rule-of-thumb” requirement in Harman’s single-factor test for potential issues with common method bias. Therefore, we conclude that our measurement model has minimal to no common method bias issues.

### Structural model

**Fig. 2:**
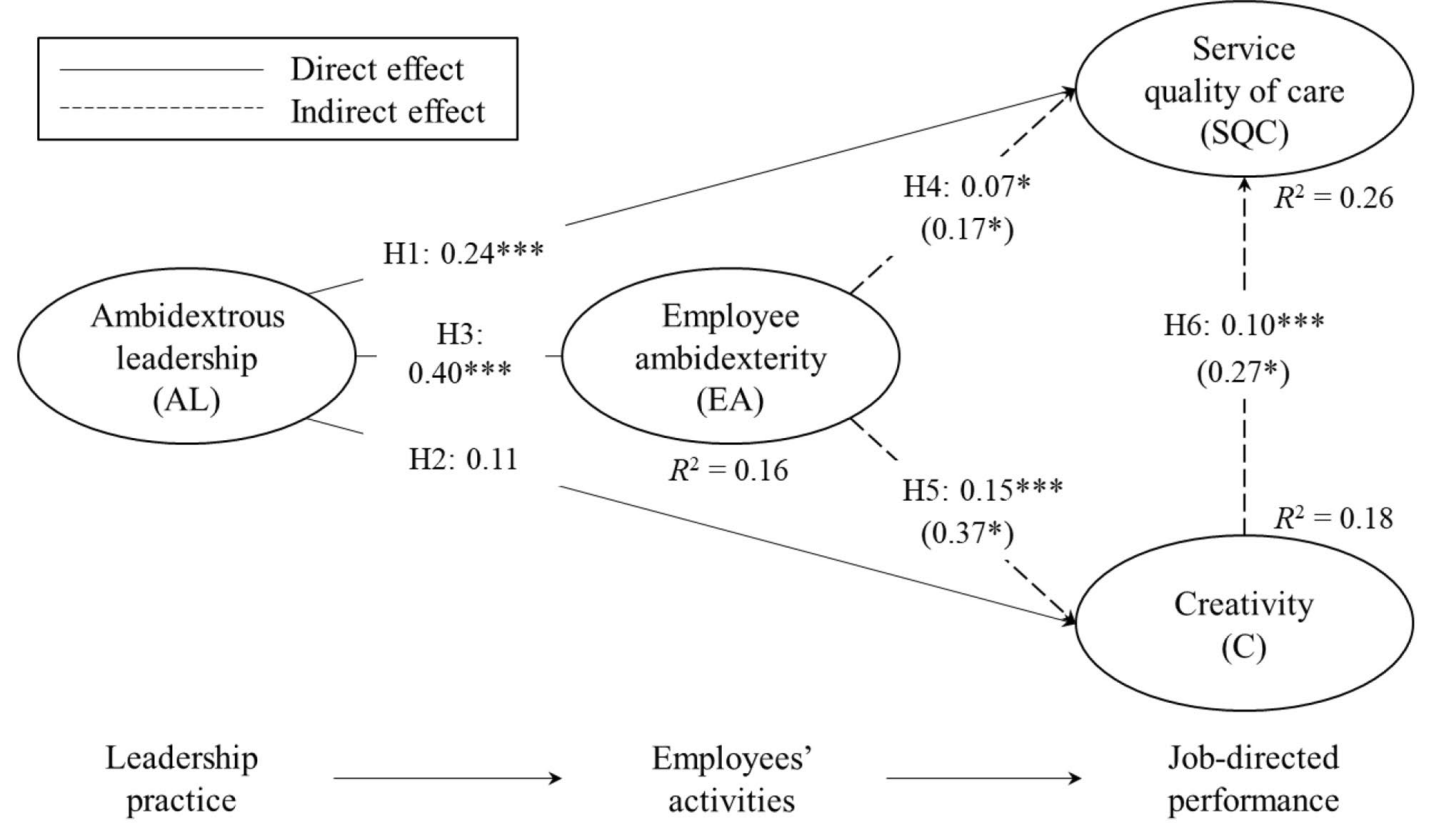
Direct and indirect effect results from the structural model of the links between AL, EA, SQC and C. Standardized coefficients (*** < 0.01, ** < 0.05, * < 0.10). Note that the reported path parameters on the dotted lines are indirect effects while the direct effect are shown in parentheses^2^

In line with Hair et al. [36] and Caniëls et al. [41], we tested for potential influences on the structural model through control variables using personal characteristics. The results were nonsignificant, and therefore, control variables are not included further in the results of this study. We further tested the intra-construct correlations of the latent variables, namely Al, C, SQC, and EA, and found no issues (see Table 4 for full details). The results for the structural model are presented in Fig. 2. For the endogenous constructs, the in-sample predictive power of the model (*R*^2^) was 0.16 for EA, 0.26 for SQC, and 0.18 for C. Based on the ‘rules of thumb’ [36], these *R*^2^ values were considered moderate.

**Table 4:** Intra-construct (latent variables) correlations

	AL	C	EA	SQC
Ambidextrous leadership (AL)	1.000			
Creativity (C)	0.256	1.000		
Employee ambidexterity (EA)	0.395	0.413	1.000	
Service quality of care (SQC)	0.372	0.401	0.372	1.000

Health professionals’ AL significantly affected their SQC, a finding that supports hypothesis 1. In detail, the results of the proposed relationship of AL and SQC showed a path coefficient β = 0.24, (p-value = 0.010). Although hypothesis 2 has a positive coefficient with a path coefficient β = 0.11, the relationship between health professionals’ AL and C is not statistically significant (p-value = 0.111). Furthermore, the results of the proposed relationship (hypothesis 3) of AL and EA showed a path coefficient β = 0.40 (p-value = 0.000). We found a significant positive association between AL and EA, which supports hypothesis 3.

We then analyzed the proposed mediation hypotheses 4, 5, and 6, and found support for all of them. In detail, the results of the proposed mediating relationship in hypothesis 4, i.e., that EA mediates the relationship between AL and SQC, showed a path coefficient β = 0.07 (p-value = 0.048). Furthermore, the results of the proposed mediating relationship in hypothesis 5, that EA mediates the relationship between AL and C, showed a path coefficient β = 0.15 (p-value of 0.002). Finally, the results of the proposed mediating relationship in hypothesis 6, that C mediates the relationship between EA and SQC, showed a path coefficient β = 0.10 (p-value = 0.003). This means that the relationship for health professionals between AL and SQC and between AL and C is mediated by EA (hypotheses 4 and 5), and the association between EA and SQC is mediated by C (hypothesis 6). In summary, we found partial mediation between the proposed relationship of hypothesis 4 and hypothesis 6 and full mediation as proposed in hypothesis 5.

## Discussion

Organizations are “collections of people joined together in pursuit of a common cause, and it is the people who create value” [42, p. 42]. Consequently, it becomes essential for organizations to discover how to create the most appropriate or “right” preconditions for how organizational members can effectively create value. This study uncovered what role AL and EA have for health professionals’ value creation manifested in their job-directed performance (referring to SQC and C) as frontline employees.

The study makes three contributions. First, it conceptualizes AL as a leadership style and practice of health professionals and contributes to understanding how AL is linked to and affects service quality in health care. Second, it proposes EA as a type of employee activity that AL affects. Moreover, the important role of AE in promoting health professionals’ SQC and C is also revealed. Third, the study reveals how and in what way health professionals’ AE and C can strengthen health professionals’ SQC offerings. This is, to the authors’ knowledge, the very first study to both conceptualize and empirically test AL and EA as factors developing the SQC and C of health professionals. The study responds to the call for research on the concept of AL [9] in health-care research. Consequently, it offers new insight into a domain that has been relatively neglected within health services research. In the following, the three main contributions are further discussed, and the main practical implications are highlighted.

### AL in health care and its link to service quality

In line with previous research, AL is defined as a leadership practice combining the duality of “opening” and “closing” leadership behaviors [11]. The findings reveal that AL is directly linked to the SQC and EA of health professionals. In addition, AL also works indirectly through EA to strengthen the SQC and C of health professionals. Consequently, these findings also illustrate AL’s multiple roles in motivating health professionals’ activities (referring to EA) and their job-directed performance (SQC and C).

When comparing all the direct effects of AL tested, it becomes clear that AL is mostly a significant driver of health professional activities represented by the concept of EA$(\beta$ = 0.395). This supports the notion that how leadership is practiced in the organization is “absorbed” by organizational members and functions to motivate and “model” behavioral orientation (EA) of these organizational members. This result is consistent with research showing that leadership influences the employees [10].

Furthermore, the close link between AL and EA reflects the truth emphasized in social learning theory [31], claiming that employees adopt and learn what is proper and allowed behavior from relevant others, such as observing the “walk and/or talk” of their leaders. As Luu noted, “[an] ambidextrous leader may serve as a role model for public employees to emulate exploratory and exploitation behaviors, leading to individual ambidexterity among public employees” [32 p. 507]. Thus, the combination of the “opening” and “closing” behavior of health-care leaders practicing AL enhances and boosts the (two) activities embraced by health professionals’ EA.

Considering the promotion of health professionals’ SQC, this study reveals the value of the manifestation of AL in two ways. First, AL was found to have a direct impact on the enhancement of SQC of health professionals $(\beta$ = 0.236). Second, AL also works indirectly to promote SQC through the mediating effect of EA. Consequently, the findings reveal two complementary paths for how to improve health professionals’ SQC. Both paths stem from AL. This is a highly interesting and important finding, especially given the absence of previous health service research regarding the quality of in-home care services [43]. Most previous research on service quality in general has been undertaken within hospital organizations [43]. This is an important context to study service quality, which is why this study adds to research in a context that has been more or less “under-researched” [43, p. 2]. It contributes to revealing AL’s role in health-care management in home-care services.

Specifically, it shows the importance for organizations to develop “opening” and “closing” behavioral leadership competency (referring to AL), as it has a positive relationship with health professionals’ SQC. At the same time, AL can nurture health professionals’ exploration and exploitation activities (or EA) that enhance health professionals’ SQC. These direct and indirect impacts (via EA) of AL find support within the job demands-resources (JD-R) framework [21]. Considering AL as a type of specific encouraging or supportive leadership behavior provided to health professionals, the results of this leadership practice are an enhancement in SQC. Consequently, the (direct and indirect) effect between AL and SQC identified points out that “leadership is important to quality” [ 1, p. 1] in in-home care services. As a practical implication, these findings highlight the value for health-care organizations of leaders with a high level of AL competencies. Thus, health-care organizations should seek to recruit leaders with AL capacity or implement training programs and exercises that improve their leaders’ AL capability.

### The important role of EA as a promoter of creativity in health care

Although the results show that AL has two routes to enhance SQC, this was not the case when testing the relationship between AL and C. The expected direct link between the AL and C of health professionals turned out to be nonsignificant. Based on interpreting results from the two patterns of relationship tested (one direct and the other indirect) between AL and C, this indicates the existence of only one way to how AL can enhance health professionals’ C, i.e., when the relationship between AL and C is mediated through EA.

According to Kim et al., “creativity… is a clear leadership priority in the modern health care organization” [44, p. 1]. No previous research has explored how AL affects health professionals’ C within health services research. Only a few research studies tested the relationship pattern between AL and C, and all were undertaken in contexts other than health care. These studies have found that AL and C are related. One example is the study of Tung [45] on 427 employees in an electronic industry in China, in which the author found AL and employees’ C to be significantly related$(\beta$ = 0.289). Another example is Zacher and Rosing’s [33] study using multi-source survey data from 33 team leaders and 90 employees in an architectural and interior design firm. The authors found support for a relationship between AL and team innovation. Team innovation in this study was defined as a combination of both generation of novel and original ideas (comparable to the definition in this study) and implementation of creative ideas. A third example is the study of Zacher and Wilden [46]. Based on diary data collected from 113 employees across five workdays, the authors found empirical support for the relationship between the high level of “open” and “closed” leadership behavior (referring to AL) and employees’ self-reported innovative performance. Innovative performance in Zacher and Wilden [46] is relatively comparable to the concept and content of C used in this study (respondents were questioned about “coming up with new ideas” and “finding improved ways to do things”).

In contrast to the studies referred to above (see [33, 45, 46]), this study did not find support for a direct linkage between AL and C. However, our study differs from the literature in three substantial ways. First, as indicated in the previous discussion, this study was undertaken in a health care setting. Second, data were based on health professionals working on the front lines. Thus, they work directly “face to face” with users of home-care services. Third—and this is probably the most essential difference—this study introduced EA as an indirect, complementary “route” by suggesting EA to be a mediator between AL and C. By testing a direct effect (of AL on C) and simultaneously an indirect effect (of AL on C via EA), the two types of routes “compete” to explain how AL operates to enhance the C of health professionals.

The findings of this study signal that EA plays a mediating role between AL and C. In this reasoning, it is also interesting to note the strength of the association between EA and C $(\beta$ = 0.370) when compared with all other associations tested in this study. This shows that AL and C have the second-strongest relationship. The strength of this linkage is almost the same as between the AL and EA $(\beta$ = 0.395), which was the strongest relationship identified in this study. The role of EA is to enhance health professionals’ C.

These findings revealed that leaders practicing AL is a necessary condition but not enough to “fuel” health professionals’ C. By contrast, the findings show that AL only impacts health professionals’ C when their EA is put to work first. According to Kim et al., leaders should be “motivated to facilitate creativity among their… frontline-staff” [44, p. 1]. Furthermore, Kim et al. also commented that it is “unclear how to most effectively achieve such outcomes.” However, this study contributes new insight and understanding, revealing that AL is a motivational and facilitating factor to enhance health professionals’ C. On the other hand, it also shows that C is only achieved by first enhancing the EA of health professionals. Consequently, the study identifies a pattern of necessary processes (EA) associated with AL that leads to achieving C as a job-directed performance. A practical implication based on the findings is that AL and EA should be prioritized when health organizations desire to cultivate the C of their health professionals. It should be explored if it is possible to run workshops that develop ambidexterity skills for leaders and employees. This could include team building or training that develops both exploratory and exploitative competencies for all employees, including leaders.

### The role of EA and creativity for service quality in health care

In their article, Schnellbächer et al. noted that “few studies have assessed the performance effects of individual ambidexterity [47, p. 443]. As shown in the previous discussion, this study empirically determines C as an outcome of EA. Thus, EA plays an important role. On the other hand, this is not limited to suggesting C as the only job-directed performance of EA. To develop employees, C does not have any real value. Only when C is perceived as a means to achieve a desired end can it be considered valuable. Consequently, from an organizational perspective, C is only (or most) interesting when there is a potential to capitalize on the employees C. C can be manifested in many ways and forms, such as enhanced productivity, a new process, routine, service and, in general, a (new) change that improves and makes positive progress compared to how and what things have been done in the past. Findings from this study indicate that there is a clear potential to capitalize on C to enhance health professionals’ SQC $(\beta$- value between C and SQC were 0.273). The three variables, C, AL, and EA, explain about 26% (25.9) of the variance in SQC. However, when comparing the three variables, the findings from this study reveal that EA has a significant role.

Specifically, when EA is nurtured because leaders practice AL, it drives employees’ C, which is next capitalized in the manifestation of positive changes in the SQC of health professionals. Thus, EA, stemming from AL, is essential in engaging health professionals in creative thinking and improving their SQC. The study contributes to revealing the important role of EA in enhancing both C and SQC. The capability of EA to energize and engage job-directed performance (C and SQC) supports Hewko’s view: “Without engaging in… creative thinking, professionals… find it difficult to identify… new ways of working” [24, p. 1]. As Rosing and Zacher remarked, we have to develop an appropriate “context that allows… [employees]… to act ambidextrously” [30, p. 696]. As shown in this study, when leaders create a context by practicing AL, the EA of health professionals is boosted. Additionally, the findings reveal that EA, as well as AL, is capable of enhancing C and SQC. Although some differences exist in their pattern, AL and EA can be considered key ingredients in promoting health professionals’ job-directed performance (C and SQC). A practical implication of this finding is the importance for leaders in health-care services to develop the organizational environment or ecosystem in such a way that it stimulates and cultivates the EA of their health professionals.

### Limitations and further research

To the authors’ knowledge, this is a pioneering study focusing on the role of AL and EA within health services research. Naturally, because of its newness, several improvements could be included to extend and deepen this study. In this section, three areas are mentioned that could be considered in future research.

First, AL and EA in this study are limited to only embracing two types of job-directed performance (SQC and C). Future research should include other potential outcomes. One concrete suggestion is to add innovation to the “list” of outcomes. Specifically, in line with participants of this study (health professionals), innovation should be studied from a frontline perspective, sometimes described in the literature as innovative work behavior [48], innovative behavior [49], and creative performance [50]. Studying employee innovativeness in a health-care context is both relevant and important. Regarding it criticality, Hewko recently stated: “Among knowledge workers, including health professionals, innovativeness is of particular importance” [24, p. 1]. However, how and in what way frontline employees’ innovativeness in health-care organizations should be stimulated is not clarified. Consequently, based on the critical role leadership has, there is a need for more research linking AL to frontline health professionals’ innovative behavior. Doing so will provide new insight into the role of AL as a potential (frontline) innovativeness promotor in health services.

Second, as mentioned in the previous discussion, it is essential to have a “context that allows… [employees]… to act ambidextrously” [30, p. 696]. A context is a mixture of numerous factors that are potentially interconnected and influence each other either positively or negatively. This study limited its focus to contextual variables for the leadership practicing AL. The findings reveal that AL, as a part of the health professional’s context, was found to be a booster for EA. However, other highly interesting context-related variables could be included in future research. One example is the relatively new concept of “thriving at work” (TAW) proposed by Spreitzer et al. [51]. Stemming from positive psychology, the concept of TAW reflects individual employees’ experiences of having a good work life when employed in an organization. TAW has been described as “essential for… a sustainable performance” [52, p. 249]. Previous research in other health-care contexts suggests that TAW mediates AL and employees’ work performance [53]. This implies the significance of leaders being aware of and understanding how to create a context where all members of organizations are TAW. It is therefore surprising, considering both the criticality of human resources and how people-intensive health-care organizations are, that only a few research studies have focused on TAW within a health-care context. Thus, there is an urgent need to include TAW more extensively in future health services research. Specifically, based on this study, it is suggested to examine the role of TAW in relation to AL, EA, SQC, and C.

Third, the dominant and one-sided view in the literature is to describe AL and EA as positive and something “all” employees perceive as uniquely good and are grateful for. We do not know much about “the other side of the coin” and if there are some potential negative characteristics related to AL and EA. Future research should examine whether there are some situations and/or circumstances, personality traits, or states where AL and EA have negative impacts on SQC and C or other potential job-related performance. For example, when leaders practice (AL) “opening” and “closing,” it may cause frustration, anxiety, tendencies to mental or physical burnout, and fear of punishment for not correctly practicing (EA) “exploration” and “exploitation.” Is there a need to have some safeguarding mechanism in place associated with AL and EA? Future research could include (mutual) trust between leaders and employees as one type of safeguarding mechanism. Another suggestion for future research is to include the concept of psychological safety as a mediating safeguarding mechanism. The study of such potentially damaging aspects associated with AL and EA could be achieved by using a quantitative method, qualitative method, or a combination of the two methodological approaches (e.g., mixed methods). This would contribute to a balanced view and a nuanced perspective about how and when AL and EA can be perceived as something “good.”

## Conclusion

This paper contributes new knowledge regarding AL and EA, which is a domain that has been neglected in previous health services research. Specifically, the findings reveal how AL and EA can promote health professionals’ SQC and C. The study implies that health-care organizations must be determined to employ, train, and expand their leaders to become AL. Doing so will have a direct impact on SQC as well as EA. In addition, AL will also transform EA in a positive direction and indirectly stimulate employees’ C as well as strengthen health professionals’ SQC offerings. Consequently, health organizations should be aware of those multiple positive “domino effects” achieved by having leaders practicing AL.

## Data Availability

The datasets used and/or analyzed in this study are available from the corresponding author upon reasonable request.

## Footnotes

1 Because it is a lengthy analysis, the full report of the results of the Harman single-factor test is available from the authors upon request.

2 To save space, we did not report all estimated parameters in the structural model and from the mediator analysis. However, these results are available from the authors upon request

## Abbreviations

SEM: Structural equation modeling
SRMR: Standardized root mean square residual
RMSEA: Root mean square error of approximation
CFI: Comparative fit index
TLI: Tucker–Lewis index
RCC: Raykov’s reliability coefficient
AVE: Average variance extracted
SQC: Service quality of care
AL: Ambidextrous leadership
EA: Employee Ambidexterity
C: Creativity
NSD: Norwegian Social Science Data Services
TAW: Thriving at work
